# Identification of potentially anti-COVID-19 active drugs using the connectivity MAP

**DOI:** 10.1371/journal.pone.0262751

**Published:** 2022-01-27

**Authors:** Raphaël Bonnet, Lee Mariault, Jean-François Peyron

**Affiliations:** 1 Université Côte d’Azur, Nice, France; 2 Inserm U1065 Centre Méditerranéen de Médecine Moléculaire (C3M), Nice, France; Wayne State University School of Medicine, UNITED STATES

## Abstract

Drug repurposing can be an interesting strategy for an emergency response to the severe acute respiratory syndrome-coronavirus-2, (SARS-COV-2), the causing agent of the coronavirus disease-19 (COVID-19) pandemic. For this, we applied the Connectivity Map (CMap) bioinformatic resource to identify drugs that generate, in the CMap database, gene expression profiles (GEP) that negatively correlate with a SARS-COV-2 GEP, anticipating that these drugs could antagonize the deleterious effects of the virus at cell, tissue or organism levels. We identified several anti-cancer compounds that target MDM2 in the p53 pathway or signaling proteins: Ras, PKBβ, Nitric Oxide synthase, Rho kinase, all involved in the transmission of proliferative and growth signals. We hypothesized that these drugs could interfere with the high rate of biomass synthesis in infected cells, a feature shared with cancer cells. Other compounds including etomoxir, triacsin-c, PTB1-IN-3, are known to modulate lipid metabolism or to favor catabolic reactions by activating AMPK. Four different anti-inflammatory molecules, including dexamethasone, fluorometholone and cytosporone-b, targeting the glucocorticoid receptor, cyclooxygenase, or NUR77 also came out of the analysis. These results represent a first step in the characterization of potential repositioning strategies to treat SARS-COV-2.

## Introduction

The severe acute respiratory syndrome-coronavirus-2, SARS-COV-2, emerged in December 2019, causing a viral outbreak declared as a pandemic by the World Health Organization (WHO) [1]. By November 2021, more than 254 million people had already developed coronavirus disease-19, COVID-19, which resulted in almost 5.1 million deaths worldwide [2]. In contrast to “common“coronaviruses that only infect the upper respiratory tract, SARS-COV-2 also replicates in the lower respiratory tract eventually causing pneumonia that can progress to an acute respiratory distress syndrome (ARDS) and in some cases lethal multiple organ failure.

While most infected people will remain asymptomatic, some will start to develop, after an incubation period of 0–14 days, a mild infection of both the upper and lower respiratory tract. The situation can worsen for 20–25% of the patients who develop a strong response to the virus and secondary bacterial/fungal infections, generating the release of inflammatory cytokines, chemokines, and alarmins by both the innate and adaptive immune systems [3]. This “cytokine storm”causes an uncontrolled inflammatory response that dramatically reduces lung function, associated with vasculopathy and coagulopathy. The combination of virus replication, immune cells attraction and this “cytokine storm” creates hypercoagulability in lung and alveoli associated with alterations to endothelial cells due to macrophages and neutrophil infiltration. SARS-COV-2’s spike protein can also damage endothelial cells by decreasing ACE2 expression [4]. This process of immunothrombosis greatly participates in the ARDS [5] that precedes symptoms of sepsis and multiple organ failure that may culminate in patients death [6].

In an intense race to stop this pandemic and its associated death toll, all possible forces must be mobilized to have a better understanding of COVID-19 pathological features in order to improve patients’ care and develop new treatments to prevent the onset of severe cases.

Vaccines have been developed at high-speed [7], especially thanks to a novel approach using mRNA to express the spike viral protein. In contrast, drug development takes too much time considering the speed of the disease. Therefore, drug repurposing appears to be a beneficial strategy [8, 9]. Several drugs with anti-viral potential have been proposed: lopinavir/ritonavir, remdesivir, hydroxychloroquine, making headlines and controversies, but only few drugs have proven to be effective. The common anti-inflammatory and immunosuppressive glucocorticoid dexamethasone appears to be the most efficient one to date [10, 11].

Besides the continuation of vaccines development, many clinical trials are still ongoing. As of november 2021, 7031 trials testing for potential anti-COVID19 drugs had been registered, including 706 active ones.

We used the CMap bioinformatics resource from the Broad Institute [12], to screen for potentially active anti-COVID-19 molecules. For this, we identified the differentially expressed genes (DEGs) from cell lines exposed to SARS-COV-2 or from infected lung tissues, compared to non-infected cell lines and healthy tissues using publicly available transcriptomic data. Then, with these genes, we queried the CMap database containing perturbagen-driven gene expression profiles (GEP) [13, 14] to identify molecules able to generate GEPs that negatively correlate with the provided COVID-19 gene signature. Such molecules are theoretically expected to antagonize or prevent the deleterious effects of the virus [15].

Among several candidate molecules, we were able to highlight anti-inflammatory molecules, anti-cancer agents, signaling and metabolite inhibitors, neuromodulators. These molecules could potentially represent new repositioning resources to fight SARS-COV-2 infection, and interestingly, some of them have already been associated with SARS-COV-2/COVID-19 literature or are being tested in COVID-19 clinical trials.

## Materials & methods

### SARS-COV-2 dataset

We used the publicly available GSE147507 dataset that studied the transcriptional response to SARS-COV-2 infection [16]. We extracted the transcriptomic data from two human cellular models: primary normal bronchial epithelial (NHBE) cells, and transformed lung-derived Calu-3 cell line, that were either mock-treated or infected with SARS-CoV-2. We did not consider the data from the A549 lung alveolar cell line because these cells were engineered to express ACE2 to become sensitive to SARS-CoV-2.

We also selected data obtained from primary human lung biopsies. Uninfected biopsies were from two individuals and used as replicates. A virus-infected biopsy corresponded to a patient deceased from COVID-19 [16].

### Bioinformatical analysis

For gene expression analysis, we used the web-application Phantasus [17]. We processed the data and identified differentially expressed genes (DEG) between pooled healthy samples (n = 8) and pooled SARS-CoV-2 infected samples (n = 8). We applied log2 and quantile normalization followed by Limma to identify 229 upregulated DEG and 162 downregulated DEG (p<0.05) (S1 Table). Using the protein-protein interaction STRING database (https://string-db.org/), we queried our top 50 upregulated genes to create a network that highlights both functional and physical protein associations. We selected interactions with a minimum required score of 0.9 (most strict confidence filter).

To identify enriched pathways from these DEG we used the R package fGSEA in the Molecular Signature Database (MSigDB: https://www.gsea-msigdb.org) and the Reactive database (https://bioconductor.org).

### Connectivity Map analysis

We ran the second version of the Connectivity Map dataset referred as “build 02”(https://portals.broadinstitute.org/cmap/) and used the Clue.io graphical interface (https://clue.io/). According to the CMap guidelines, we used the top 150 up- and down-regulated DEGs. Compounds identified with the CMap algorithm were filtered based on their enrichment scores (ES, Score<-90). We selected molecules/perturbagens with negative ES, meaning that they reversed the SARS-COV-2 DEG signature and as a consequence could harbor anti-COVID-19 properties. The 22 molecules with the highest negative ES are presented and discussed in this study.

### Ethic statement

This research is based on publicly available human transcriptomic data.

## Results

### SARS-COV-2 induces a transcriptional immune response

**Fig 1:**
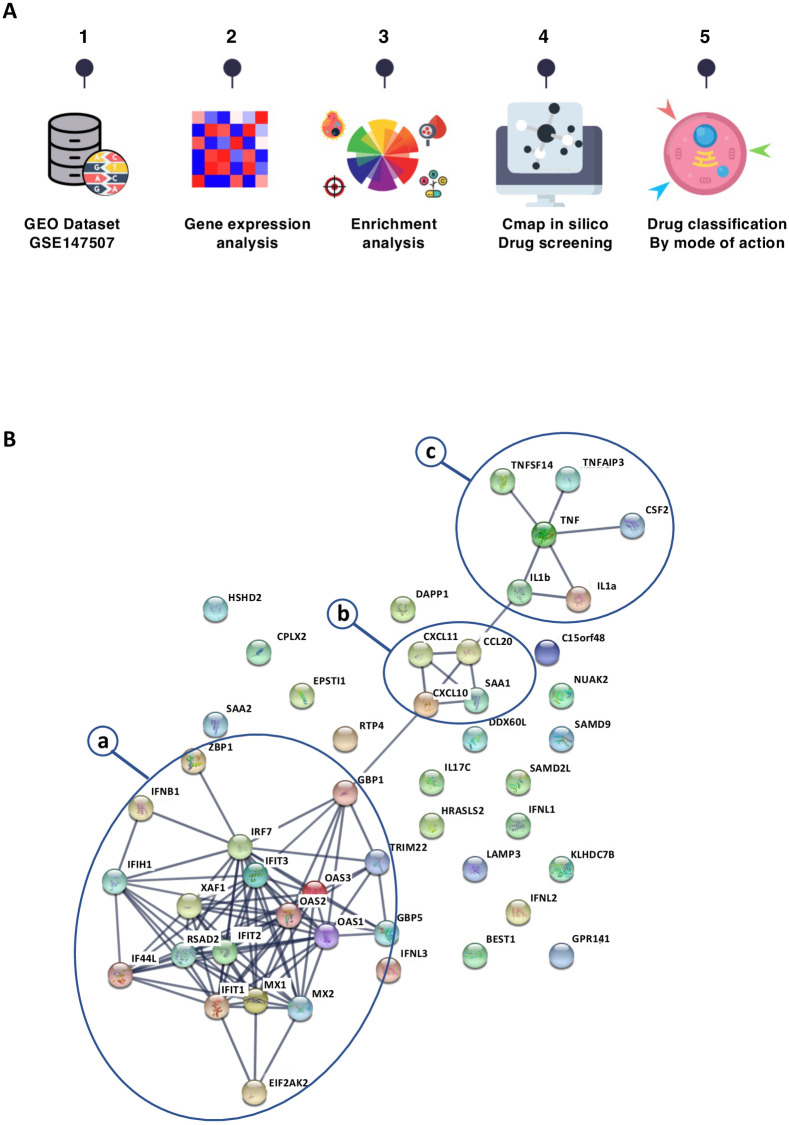
A: Workflow of the bioinformatic analysis. B: Using the protein-protein interaction STRING database, we queried our top 50 upregulated genes (sorted by LogFC). The network edges indicate both functional and physical protein associations, the line thickness represents the confidence, it indicates the strength of data support with a minimum required interaction score of 0.9 (most strict confidence filter). Three modules (a: interferon anti-viral proteins, b: interferon-induced chemokines, c: TNF and inflammatory cytokines) are encircled.

As shown in our experimental workflow (Fig 1A), we started our analysis by selecting transcriptomic data from the publicly available GSE147507 dataset that was one of the first studies on SARS-COV-2 to become public in March 2020. It characterizes the transcriptional response to SARS-COV-2 in a variety of model systems, *in vitro* and *in vivo* [16]. We selected transcriptomic data that correspond to a SARS-COV-2 infection of human primary normal bronchial epithelial (NHBE) cells, and of cells from the transformed-lung-derived Calu-3 cell line. In addition, the dataset contained RNAseq data obtained from lung biopsies of a COVID-19-deceased patient and of two uninfected individuals. We determined the differentially expressed genes (DEGs) between pooled uninfected and pooled SARS-COV-2 infected samples (S1 Table). This resulted in the identification of 229 upregulated and 162 downregulated DEGs (p<0.05). These DEGs were then analyzed with the fGSEA R package to identify enriched pathways using the MSigDB molecular signature database and the Reactome databases (S2 Table). Unsurprisingly, SARS-COV-2 infection was found associated with the up-regulation of genes involved in innate and adaptive immune responses. We observed positive enrichment for interferons, chemokines, and interleukins involved in anti-viral responses (S1 Fig). We used a network-based approach to highlight the functional and physical interactions between our top 50 upregulated genes (Fig 1B). In this network, the largest module of connected genes (a) includes interferon beta and interferon-induced genes coding for the anti-viral proteins IFT1, IFIT2, IFIT3, OAS1, OAS2, OAS3, MX1, MX3, TRIM22. Another module (b) is composed of three interferon-inducible chemokines: CXCL10, CXCL11, CCL20 involved in infectious responses and the recruitment of immune cells. Serum amyloid A1 (SAA1) which exerts pleiotropic immunomodulatory functions is also connected to these chemokines. Despite this interferon response, it was shown by the authors of the study [16], that the SARS-COV-2 virus is a low inducer of the IFN-I and IFN-III systems, a failure which likely contributes to the development of COVID-19 during the viral infection.

A third module (c) revolves around TNF and includes IL1A, IL1B, TNFSF14, TNFAIP3, and CSF2 that likely mediates the inflammatory response associated with COVID-19 progression. It is conceivable that these genes could be used as biomarkers to evaluate the effects of potential anti-COVID-19 drugs.

### A Connectivity Map analysis identifies potential anti-COVID-19 compounds

**Fig 2:**
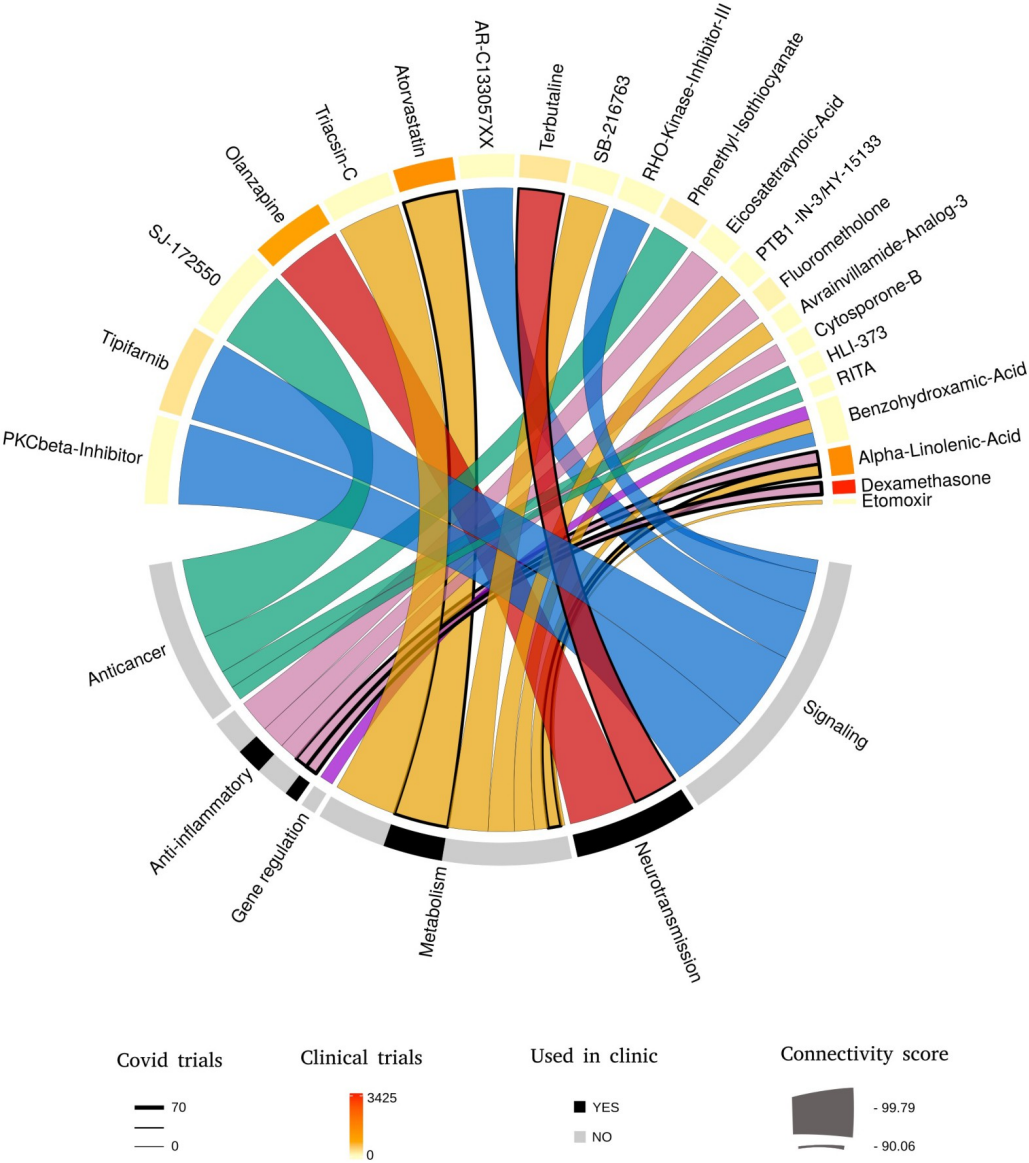
Using the *circlize* R package, the most significant CMap results (score >90) are represented in this figure. On the top part, compounds are ordered clockwise based on their enrichment score and are classified on the bottom part according to their mode of action. Additional information on their use in clinical trials and in therapies are present through upper orange and lower black gradients, respectively. The ribbons borders indicate the number of covid trials that have been already conducted on each molecule.

We ran a Connectivity Map analysis using the top 150 up- and down-regulated genes. After filtering based on the enrichment scores of compounds (Score<-90), we selected 22 compounds displaying the highest negative enrichment score (Table 1). A negative ES reflects the capacity of a molecule to produce a gene signature that negatively correlates or reverses the input signature. Here, by reversing a gene signature associated with COVID-19, it is expected that the candidate molecules could counteract the damaging effects of the SARS-COV-2 virus. The 22 selected compounds that are presented on a Circos plot (Fig 2) distribute in 5 categories: signaling inhibitors (n = 5), anticancer drugs (n = 4), neurotransmission modifiers (n = 2), metabolism-regulating compounds (n = 8), anti-inflammatory drugs (n = 5) (S3 Table). One compound, benzohydroxamic acid, was difficult to classify because of its poorly detailed and diverse mode of action (MOA) ranging from anti-bacterial and anti-fungal to enzyme regulator and modifier of gene expression through inhibition of histone deacetylation.

**Table 1:** Results of the CMap analysis. The compounds are classified according to their score.

Rank	Score	Type	ID	Name	Description
8558	‒99.79	cp	BRD-K63195589	tipifarnib	Farnesyltransferase inhibitor
8559	‒99.79	cp	BRD-K89687904	PKCbeta-inhibitor	PKC inhibitor
8557	‒99.72	cp	BRD-K93095519	SJ-172550	MDM inhibitor
8542	‒98.23	cp	BRD-K18895904	olanzapine	Dopamine receptor antagonist
8533	‒97.62	cp	BRD-K80527266	triacsin-c	Adrenergic receptor antagonist
8528	‒96.53	cp	BRD-U88459701	atorvastatin	HMGCR inhibitor
8525	‒95.98	cp	BRD-K40892394	AR-C133057XX	Nitric oxide synthase inhibitor
8523	‒95.34	cp	BRD-A50157456	terbutaline	Adrenergic receptor agonist
8517	‒94.72	cp	BRD-K59184148	SB-216763	Glycogen synthase kinase inhibitor
8515	‒94.54	cp	BRD-K23875128	RHO-kinase-inhibitor-III[rockout]	Rho associated kinase inhibitor
8514	‒94.53	cp	BRD-K56700933	phenethyl-isothiocyanate	Antineoplastic
8508	‒93.73	cp	BRD-K06080977	eicosatetraynoic-acid	Cyclooxygenase inhibitor
8500	‒92.92	cp	BRD-K16554956	PTB1	AMPK activator
8497	‒92.59	cp	BRD-A13133631	fluorometholone	Glucocorticoid receptor agonist
8495	‒92.11	cp	BRD-K39569857	avrainvillamide-analog-3	nucleophosmin inhibitor
8493	‒91.99	cp	BRD-K86191271	cytosporone-b	NUR77 receptor agonist
8491	‒91.88	cp	BRD-K17349619	HLI-373	MDM inhibitor
8484	‒91.36	cp	BRD-K00317371	RITA	MDM inhibitor
8483	‒91.28	cp	BRD-K59773493	benzohydroxamic-acid	Antifungal
8482	‒91.19	cp	BRD-K33396764	alpha-linolenic-acid	Omega 3 fatty acid stimulant
8479	‒91.07	cp	BRD-A35108200	dexamethasone	Glucocorticoid receptor agonist
8467	‒90.06	cp	BRD-K77625572	etomoxir	Carnitine palmitoyltransferase inhibitor

As the CMap database is composed of transcriptional signatures that have been generated on different cellular models by molecules/perturbagens, some being already FDA-approved or involved in pre-clinical studies, it is not surprising that our selection contains five compounds: olanzapine, terbutaline, atorvastatin, fluorometholone and dexamethasone that are already used in clinic and still investigated in various clinical trials (S3 Table). Two trials concern COVID-19. For instance, dexamethasone is used to decrease the inflammation-induced lung injury in COVID19 patients [11]. Atorvastatin, the lipid-lowering agent is tested as adjunctive therapy, in combination with anti-coagulants and to alleviate the long-term effects of COVID-19 in several trials. Other compounds are interfering with diverse cellular functions. Several drugs are interfering with signal transduction and cell metabolism. Four drugs have anticancer properties, three being potential p53 activators. Two drugs affect dopamine and adrenergic receptors.

### Analysis of potential drug targets for COVID-19

Each identified putative active compound is associated with a specific MOA and a cellular target. The knowledge of these two parameters could shed interesting light on the mechanisms involved in the cellular responses to the SARS-COV-2 virus and the COVID-19 disease. Several compounds target molecules/enzymes that transmit activation signals. The farnesyl transferase enzyme is important to modify the function of many cellular proteins including Ras that mediates proliferation signals. PKCbeta and ROCK1 transmit activation signals in particular for immune cells. The nitric synthase is regulating the levels of the nitric oxide (NO) gaseous signaling molecule that exerts autocrine and paracrine actions, particularly on endothelial cells. Three compounds, with different chemical structures appear to target the p53 regulator MDM suggesting it could be an interesting target. The cytochrome P450 2E1 (CYP2E1) metabolizes many polar molecules, not only clearing carcinogens but also producing naturally signaling molecules such as 20-Hydroxyeicosatetraenoic acid (20-HETE) that acts on the vascular system. Several compounds are known to interfere with the metabolism, in particular fatty acid metabolism, by acting through inhibition of fatty acid synthase (FASN), carnitine palmitoyltransferase (CPT-1), glycogen synthase (GSK-3), protein tyrosine phosphatase N1 (PTPN1) to stimulate the AMPK catabolic pathway. An interesting target is nucleophosmin (NPM1) a nucleolar ribonucleoprotein not only involved in the biogenesis of ribosomes but also in cell division, chromatin remodeling, DNA repair.

Several anti-inflammatory compounds not only highlight the role of the glucocorticoid receptor but also put attention on the cyclooxygenase and the NUR77 receptor, respectively involved in the production of inflammatory prostaglandins, and in the balance between proliferation, inflammation and apoptosis.

## Discussion

The development of vaccines and drugs against an emerging virus is a several-year-long process. The repurposing of already available and clinically active drugs appears to be a valuable parallel strategy in an emergency scenario.

The CMap database of perturbagen-induced gene signatures was developed to discover potentially active compounds using gene expression profiles (GEPs) associated with disease states [12]. As most of the perturbagens are FDA-approved molecules with known dosage and toxicity profiles, repurposing can be envisioned avoiding costly drug development and lengthy phase I/II trials. The Connectivity Map (Map) developed by the Broad Institute has been used to identify drugs with the potential to interfere with diverse pathological situations such as epilepsy [18], pulmonary arterial hypertension [19], asthma [20]. Based on cancer GEPs, several molecules have been identified *in silico* and showed promising properties *in vitro*/*in vivo* in a variety of cancers such as renal cancer [21], colorectal cancer [22], bladder cancer [23], leukemia [24], neuroblastoma [25].

We used CMap on SARS-CoV-2 published signatures obtained from human lung cell lines infected with the SARS-CoV-2 virus and from primary lung biopsies from a COVID-19 deceased patient to obtain 22 potential drug candidates representing six functional categories.

Our selection identified four anticancer drugs. It has been assumed that virus-infected cells, similarly to cancer cells, are in an anabolic state reflecting their intense biomass synthesis and could therefore be sensitive to anti-cancer agents [26]. None of these four compounds was already described in the literature to interfere with SRAS-COV-2/COVID-19. Nevertheless, the fact that three structurally unrelated MDM2 inhibitors have been selected suggest that this target could be relevant. Most viruses inactivate the p53 pathway by a large variety of mechanisms, to prevent a p53-induced stress response leading to apoptosis, see for review [27]. Although the mechanisms of action of SARS-COV-2 on inactivating p53 have not been defined yet they could involve an MDM2-independent degradation of p53 [27]. Therefore, inhibiting MDM2 during SARS-COV-2 infection could slow down the disappearance of p53 by these two degradative routes, attenuating the effect of the virus. The fourth compound of this category, phenethyl-isothiocyanate (PEITC) is a phytochemical, multifaceted agent, that was shown to interfere with various cancer-promoting mechanisms through anti-inflammatory, anti-oxidant and anti-proliferative properties and had been tested in clinical trials, reviewed in [28]. Its inhibitory action on the cytochrome P450, CYP2E1, could decrease the production of the 20-HETE mediator that exerts many effects on the vascular system.

Four signaling inhibitors may have been selected for their capacity to block growth and division signals. They represent an interesting category as two of them have already been studied for COVID-19. The farnesyl transferase inhibitor of farnesyl transferase tipifarnib, appeared in a virtual screening of 6218 FDA-approved drugs, and was able to block SARS-COV-2 replication in Vero cells [29]. Whether tipifarnib acts through its attributed cellular target or directely on the virus and its replicative machinery remains to be further explored. Inhibitors of RHO-Kinase 1 (ROCK1) have been proposed to have beneficial effects against the severe acute respiratory distress syndrome (ARDS) associated with COVID-19 [30] as ROCK1 contributes to a burst in inflammation leading to ARS. Specific inhibitors of PKCbeta-RACK1, involved in immune cell activation, could help control an exacerbated immune response [31]. The nitric oxide synthase is also an interesting target but it will have to be determined if lowering NO levels to limit inflammation will surpass a decrease in the anti-viral properties of NO [32].

Our CMap analysis highlighted several drugs known to affect cell metabolism. They represent an interesting approach as infected cells adapt and stimulate their metabolism to their viral needs. Triascin-C, an inhibitor of fatty acid synthase (FASN) has been shown to interfere with the replication of SARS-COV-2 in Vero E6 cells [33]. A second drug in our list, etomoxir, can affect lipid metabolism as an inhibitor of fatty acid oxidation. Atorvastatin, a lipid-lowering drug was shown to inhibit virus entry into a lung organoid model [34]. It was selected from a CMap analysis applied to the transcriptomic data of ACE2-transduced A549 adenocarcinomic human alveolar basal epithelial cells infected by SARS-COV-2 described by [16]. The common MOA of these three drugs on lipid metabolism suggests that this function could be crucial for the virus. For instance, lipids are important for the formation of the viral envelope. Another candidate, alpha-linoleic acid, an essential fatty acid which is not used in clinic is nevertheless tested as a supplement in COVID-19 trials, despite an unclear mechanism of action but because it is a precursor for eicosapentaenoic acid (EPA) and docosahexaenoic acid (DHA), two omega-3 insaturated fatty acids with health beneficial effects. This functional category also contains PTP1B-IN-3/HY-15133, an AMPK activator that exerts catabolic functions likely to counteract the anabolic state induced by the virus in infected cells. Along the same line, the anti-diabetic drug metformin, that lowers circulating glucose and insulin levels and activates AMPK, is associated with a reduced mortality for treated diabetic COVID-19 patients, see [35] for review. Metformin is one of the anti-COVID candidate drug in another CMap approach [36]. Our selection included the SB-216763 glycogen synthase kinase inhibitor. Interestingly, GSK3-β is activated in SARS-COV-2 infected cells to promote excessive oxidant stress. It has been therefore discussed to use specific inhibitors to dampen the detrimental effects of its abnormal activation [37].

Finally, NPM1 inhibitors, such as avrainvillamide-analog-3, have been proposed as a broad anti-viral therapy because NPM1 interacts with various viral proteins in different cellular compartments to support virus replication [38]. NPM1 that exerts pleiotropic and trophic roles is hijacked by transforming mechanisms to sustain the growth of cancer cells [39].

As expected from the physiopathology of COVID-19, the CMap analysis uncovered four anti-inflammatory molecules, including dexamethasone, which has been proven to be effective in reducing mortality for patients under respiratory support [11]. Dexamethasone is currently involved in 94 COVID-19-related clinical trials to better assess its dosage, compare it to other anti-inflammatory molecules (prednisolone) or combine it with other drugs (Rendesivir, baricitinib, atazanavir…). Fluorometholone is another glucocorticoid currently implicated in 34 clinical trials, although none are related to COVID-19. An interesting compound is cytosporone-b/Csn-B, an agonist of the anti-inflammatory NUR77 orphan receptor. Indeed, it has been demonstrated that Csn-B can control virus infection and improve lung functions in influenza-infected mice by stimulating type 1 IFN production by alveolar macrophages [40]. Csn-B decreases neutrophil infiltration of the lungs to reduce inflammation. In a murine arthritis model, Csn-B has a protective effect that could be due to an increased circulation of T_regs_ [41].

Our analysis also highlighted terbutaline, an agonist of adrenergic receptors with lung bronchus dilatation properties. Terbutaline improved the condition of COVID-19 patients with asthma. Moreover, as it is used via subcutaneous injection, it presents a lower risk of virus dispersion compared to aerosol-based rebreathers [42]. Psychotropics drugs such as the olanzapine dopamine receptor antagonist, have been shown to decrease the worsened immune response and ARDS [43].

Applying CMap to COVID-19 data has already allowed different research groups to discover many potentially active molecules that could not only interfere with virus replication but also counteract its damaging effects in infected tissues. The results we present here are a first step in the path to discovery of active new drugs to target the dysregulated mechanisms of cell growth, metabolism and proliferation in SARS-COV-2-infected cells.

## Supporting information

S1 Fig Using the *fgsea* package, all the significant (pval>0.05) pathways identified within the GO and Reactome databases are represented. (PDF)

S1 Table Results of the differential gene expression analysis from the GSE147507 gene, using Phantasus. (XLSX)

S2 Table Enrichment analysis of the COVID19 -GSE147507 DEG signature using fgsea and MSigDB, Reactome databases. (XLSX)

S3 Table Classification of the 22 selected compounds. (XLSX)
