# Supplementary figures and images for: Identification of potentially anti-COVID-19 active drugs using the connectivity MAP
Source: PLoS One. 2022 Jan 27;17(1):e0262751. doi: 10.1371/journal.pone.0262751 (PMC8794112; doi:10.1371/journal.pone.0262751)

**A**

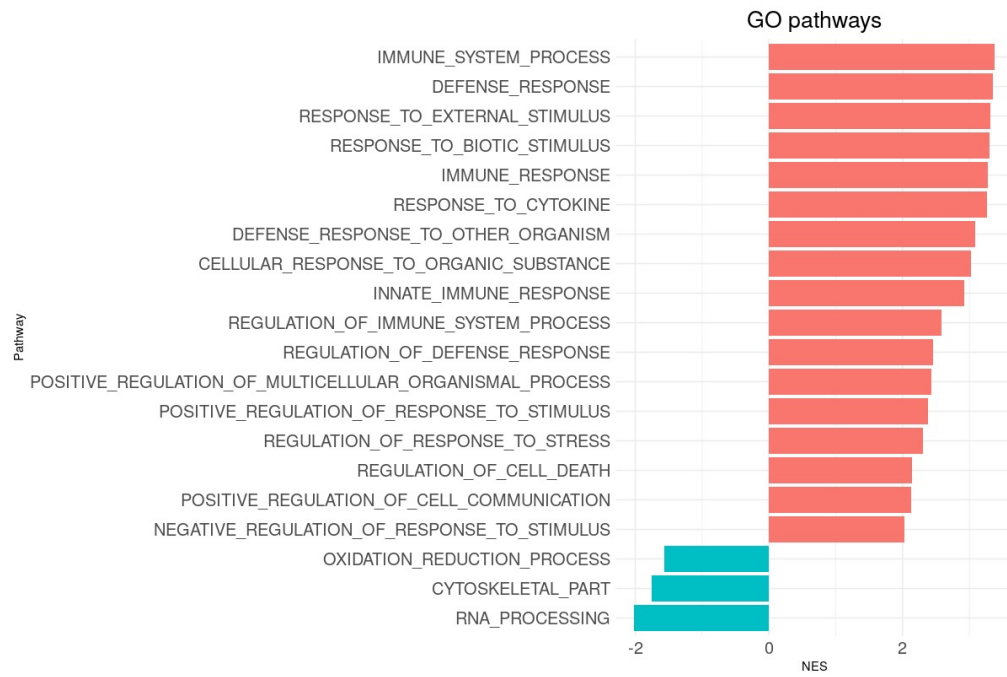

**B**

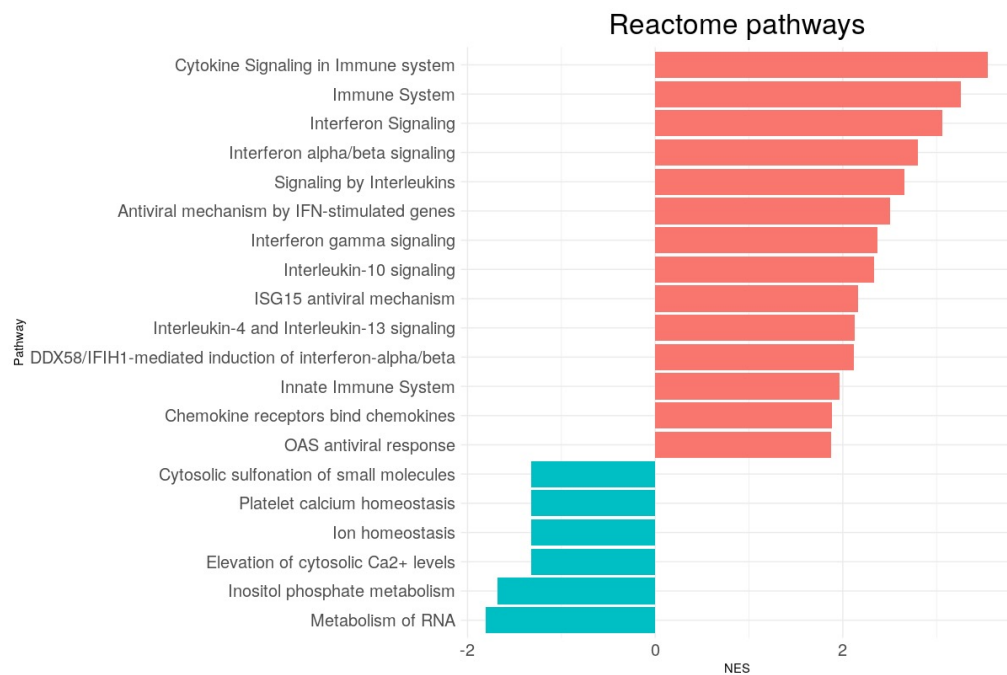

Supplement: S1 Fig — (PDF) [file pone.0262751.s001.pdf]
